# Blood pressure level impacts risk of death among HIV seropositive adults in Kenya: a retrospective analysis of electronic health records

**DOI:** 10.1186/1471-2334-14-284

**Published:** 2014-05-22

**Authors:** Gerald S Bloomfield, Joseph W Hogan, Alfred Keter, Thomas L Holland, Edwin Sang, Sylvester Kimaiyo, Eric J Velazquez

**Affiliations:** 1 Duke Clinical Research Institute, Duke University, 2400 Pratt Street, Durham, NC 27705, USA; 2 Division of Cardiology, Duke University Hospital, Suite 7400, Durham, NC 27705, USA; 3 Duke Global Health Institute, Trent Hall, 310 Trent Drive, Durham, NC 27710, USA; 4 Department of Biostatistics, Brown University, 121 S. Main Street, Providence, RI 02912, USA; 5 Academic Model Providing Access to Healthcare, PO Box 4606, Eldoret 30100, Kenya; 6 Department of Medicine, School of Medicine, College of Health Sciences, Moi University, PO Box 4606, Eldoret 30100, Kenya; 7 Division of Infectious Diseases & International Health, Department of Medicine, Duke University School of Medicine, DUMC 102359, Durham, NC 27710, USA

**Keywords:** Blood pressure, HIV, Mortality, Global health, sub-Saharan Africa

## Abstract

**Background:**

Mortality among people with human immunodeficiency virus (HIV) infection is increasingly due to non-communicable causes. This has been observed mostly in developed countries and the routine care of HIV infected individuals has now expanded to include attention to cardiovascular risk factors. Cardiovascular risk factors such as high blood pressure are often overlooked among HIV seropositive (+) individuals in sub-Saharan Africa. We aimed to determine the effect of blood pressure on mortality among HIV+ adults in Kenya.

**Methods:**

We performed a retrospective analysis of electronic medical records of a large HIV treatment program in western Kenya between 2005 and 2010. All included individuals were HIV+. We excluded participants with AIDS, who were <16 or >80 years old, or had data out of acceptable ranges. Missing data for key covariates was addressed by inverse probability weighting. Primary outcome measures were crude mortality rate and mortality hazard ratio (HR) using Cox proportional hazards models adjusted for potential confounders including HIV stage.

**Results:**

There were 49,475 (74% women) HIV+ individuals who met inclusion and exclusion criteria. Mortality rates for men and women were 3.8 and 1.8/100 person-years, respectively, and highest among those with the lowest blood pressures. Low blood pressure was associated with the highest mortality incidence rate (IR) (systolic <100 mmHg IR 5.2 [4.8-5.7]; diastolic <60 mmHg IR 9.2 [8.3-10.2]). Mortality rate among men with high systolic blood pressure without advanced HIV (3.0, 95% CI: 1.6-5.5) was higher than men with normal systolic blood pressure (1.1, 95% CI: 0.7-1.7). In weighted proportional hazards regression models, men without advanced HIV disease and systolic blood pressure ≥140 mmHg carried a higher mortality risk than normotensive men (HR: 2.39, 95% CI: 0.94-6.08).

**Conclusions:**

Although there has been little attention paid to high blood pressure among HIV+ Africans, we show that blood pressure level among HIV+ patients in Kenya is related to mortality. Low blood pressure carries the highest mortality risk. High systolic blood pressure is associated with mortality among patients whose disease is not advanced. Further investigation is needed into the cause of death for such patients.

## Background

In North America and Europe, cardiovascular disease is the second most common cause of death among HIV seropositive (+) individuals after acquired immunodeficiency syndrome (AIDS)-related mortality [1]. This trend has been accompanied by a shift in the long-term care of HIV+ patients to include attention to cardiovascular risk [2,3]. Observational studies from around the globe support the association between HIV infection and increased risk of cardiovascular disease, however, these studies have largely not included patients from developing countries [4-7].

Most people with human immunodeficiency virus (HIV) infection live in sub-Saharan Africa (SSA). Sixty-nine percent (23.5 million of 34 million) of all people infected with HIV worldwide and the majority of HIV-related deaths are in SSA [8]. Infectious and immunological factors have usually been associated with the highest risk of death in people living with HIV in SSA [9,10]. However, HIV+ individuals without AIDS in SSA can now achieve a near normal life expectancy and hypertension is not uncommon [11,12]. The degree to which high blood pressure is related to mortality in HIV+ patients in the region has not been specifically addressed [10]. This may be related to under-recognition of the magnitude of the risk of death associated with cardiovascular risk factors among HIV+ individuals in SSA.

The relationship between blood pressure level and mortality in HIV+ patients may have important policy implications as HIV+ individuals age and programs in SSA expand their scope to address the important overlap between communicable and non-communicable diseases [13]. Therefore, the aim of this study was to describe the relationship between HIV infection, blood pressure level, and death in a cohort of HIV+ adults without AIDS from a large HIV treatment program in western Kenya. By so doing, our objective was to describe the overall relationship and identify important mortality differences according to gender and clinical stage of HIV.

## Methods

### Ethics statement

This was a retrospective analysis of de-identified electronic medical records. Individual informed consent was not obtained. The Institutional Research and Ethics Committee of the Moi University School of Medicine and the Institutional Review Boards of Indiana, Duke, and Brown Universities approved use of these data and waiver of informed consent.

### Study design and participants

This retrospective study used de-identified data from the electronic medical records of HIV+ adult patients treated in the Academic Model Providing Access to Healthcare (AMPATH) program. The AMPATH program is a clinical care program based on a collaboration between Moi Teaching and Referral Hospital, Moi University School of Medicine and a consortium of North American universities. To date, AMPATH provides HIV care and treatment to >150,000 adults and children living with HIV/AIDS in over 50 clinics and satellite sites throughout western Kenya. The AMPATH clinical care system was created in 2001 and has been described in more detail previously [14]. Clinic visits occurred monthly for all patients on anti-retroviral therapy (ART) unless alternative arrangements were made with their health care provider. Patients who were not yet eligible for treatment were seen monthly or bi-monthly depending on their immunologic status and other factors in their health profile. Standard paper data collection forms were used at enrollment to the program and at each subsequent visit. Data from these forms were entered into an electronic medical record by data entry technicians. A nurse measured blood pressure during a clinical encounter using a manual or digital sphygmomanometer. These data were obtained for clinical care and were not routinely performed according to a protocol. Baseline blood pressure was used for this analysis. Height and weight were also measured as part of routine HIV care. Mortality was assessed according to data available in the electronic medical record and was statistically adjusted using a previously validated approach in the AMPATH program [15].

**Figure 1:**
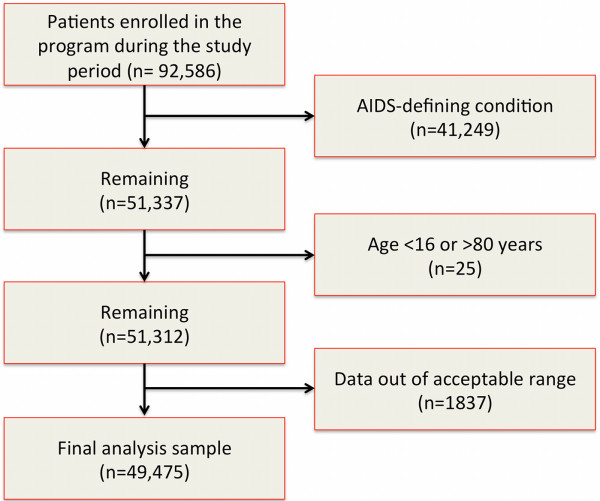
**Flow diagram of exclusion criteria to arrive at the final analysis profile.** Legend: The records of all patients enrolled in the Academic Model Providing Access to Healthcare program were screened for inclusion and exclusion criteria as described in the text. There were 92,586 patients who were enrolled in the program between January 1, 2005 and December 31, 2010. We excluded patients sequentially if they had an AIDS-defining condition, were <16 years, >80 years or had data that were out of acceptable range. Our final analysis sample included 49,475 patients.

We included HIV+ patients aged 16 to 80 years who were enrolled between January 1, 2005 and December 31, 2010 and who did not meet AIDS-defining criteria. AIDS was defined by incorporating criteria from the World Health Organization (WHO) [16] and Centers for Disease Control [17] which include CD4 count less than 200/mm^3^, any AIDS defining illness, WHO Stage 4 disease, or a diagnosis of tuberculosis at the time of enrollment or during follow-up. There were 92,586 adult patients who enrolled in the AMPATH program during the specified time period. Of these, we excluded 41,249 who had a documented AIDS-defining condition and another 25 who were younger than 16 or older than 80 at the time of enrollment. Another 1837 were excluded due to having data considered out of acceptable range using the following criteria: systolic blood pressure (SBP) <65 or >220 mmHg, diastolic blood pressure (DBP) <40 or >120 mmHg, body mass index (BMI) <15 or >40 kg/m2, and creatinine <8.84 or >884 mmol/L. There were 49,475 individuals eligible and available for analysis (Figure 1).

### Statistical analysis

The primary objective of our analyses is to characterize the association between mortality and blood pressure at enrollment. The first part of our analysis summarizes, for all individuals with available data, crude mortality rates (deaths per 100 person years of observation) across SBP (<100, 100-119, 120-139, ≥140 mmHg) and DBP categories (<60, 60-79, 80-89, ≥90 mmHg), separately by gender. In addition, we used proportional hazards regression models to characterize the conditional (adjusted) effect of SBP and DBP on mortality, adjusting for baseline demographic and clinical factors associated with blood pressure, mortality, or both. The clinical factors are CD4 cell count (200-350, >350), WHO Stage (1, 2 or 3), BMI (<18.5, 18.5-25, 25-30, >30 kg/m^2^), hemoglobin (in mg/dL), and serum creatinine level (in mmol/L). Demographic factors are age (<35, 35-44, 45-54, ≥55 years), sex, marital status (married or living with partner vs. not), and clinic location (urban vs. rural). We subdivided the sample according to those with advanced HIV disease, defined as having CD4 < 350 or WHO Stage 2 or 3. The adjusted effects of SBP and DBP are computed separately for each of the four strata defined by distinct combinations of gender and severity of HIV disease. We fit a single model, using appropriately coded indicator variables and interactions, to estimate the effects of SBP and DBP on mortality within the four strata defined by sex-HIV disease severity combinations. The adjustment variables included in the model are assumed to have the same effect across the strata. This assumption was checked using Wald tests for interactions with gender and HIV disease severity, none of which indicated evidence for including interactions.

### Handling missing covariate information

In the sample used for regression modeling, 30,224 of 49,475 individuals in the analysis sample had complete information on all of the covariates listed above. To address potential biases introduced by missing covariates, we fit the model under different assumptions about the relationship between mortality rate and having one or more missing covariates. Specifically, we use an inverse probability weighting (IPW) method that, under certain assumptions detailed in Additional file 1 (Technical appendix), corrects for potential biases attributable to differential survival distributions between those with and without missing covariates [18]. The IPW method alleviates bias due to differential survival between those with and without missing covariates. The survival distributions between those with fully observed and partially observed covariates was similar after applying the weighting, suggesting that a substantial amount of selection bias is reduced in the weighted sample, and supporting the use of IPW for fitting the proportional hazards regression (see Additional file 1).

## Results

Baseline descriptive characteristics are shown in Table 1. Our population sample (n = 49,475) had more than 74% women. Although no one in the sample had AIDS, the HIV disease burden was still substantial, with 47% of men and 37% of women having a CD4 cell count below 350, and 25% of men and 16% of women having WHO Stage 3 disease. Twenty-six percent of men and 17% of women had a BMI less than 18.5 kg/m^2^. Nearly all individuals were ART naïve at the time of enrollment (91%), however, the majority were prescribed ART at some point during the observed follow up period. With regard to specific antiretroviral agents, the most common regimen consisted of a non-nucleoside reverse transcriptase inhibitor plus two nucleoside reverse transcriptase inhibitors. Overall, 7819 (16%) were prescribed efavirenz and 19,134 (39%) were prescribed nevirapine. Protease inhibitors were prescribed for 5773 (12%) of patients. Tenofovir was used by 4047 (8%) patients and zidovudine by 15,178 (31%). Hemoglobin and creatinine were within the normal range for most patients. Both SBP and DBP were found to be in the normal range (110-119 and 60-79 mmHg, respectively) for 39% of men and 46% of women. SBP ≥140 or DBP ≥90 mmHg was found in 10% of men and 7% of women, respectively.

**Table 1:** Summary of characteristics by gender

**Variable**	**Women (n = 36616)**	**Men (n = 12859)**	**Overall (n = 49475)**
Age, median (IQR), years	32 (26-39)	38 (31-46)	33 (27-41)
Age category, No. (%), years			
<25	7473 (20.4)	812 (6.3)	8285 (16.8)
25-34	15227 (41.6)	4183 (32.5)	19410 (39.2)
35-44	9049 (24.7)	4362 (33.9)	13411 (27.1)
45-54	3739 (10.2)	2447 (19.0)	6186 (12.5)
55-64	948 (2.6)	803 (6.2)	1751 (3.5)
≥65	180 (0.5)	252 (2.0)	432 (0.9)
SBP, median (IQR), mmHg	110 (100-120)	110 (100-120)	110 (100-120)
DBP, median (IQR), mmHg	70 (60-72)	70 (60-79)	70 (60-74)
SBP category, No. (%), mmHg			
<100	3822 (10.4)	1083 (8.4)	4905 (9.9)
100-119	21145 (57.8)	6444 (50.1)	27589 (55.8)
120-139	10145 (27.7)	4488 (34.9)	14633 (29.6)
≥140	1504 (4.1)	844 (6.6)	2348 (4.8)
DBP category, No. (%), mmHg			
<60	1748 (4.8)	571 (4.4)	2319 (4.7)
60-79	27702 (75.7)	9107 (70.8)	36809 (74.4)
80-89	5905 (16.1)	2620 (20.4)	8525 (17.2)
≥90	1261 (3.4)	561 (4.4)	1822 (3.7)
BMI, median (IQR), kg/m^2a^	21.5 (19.3-24.0)	20.1 (18.4-21.9)	21.0 (19.0-23.5)
BMI category, No. (%), kg/m^2a^			
<18.5	5348 (16.8)	2855 (25.9)	8203 (19.1)
18.5 - <25	20645 (64.8)	7495 (67.9)	28140 (65.6)
25 - <30	4716 (14.8)	595 (5.4)	5311 (12.4)
≥30	1173 (3.7)	97 (0.9)	1270 (3.0)
Hemoglobin, median (IQR), g/dL^b^	11.8 (10.2-13.1)	13.8 (11.9-15.3)	12.2 (10.5-13.7)
Creatinine, median (IQR), μmol/L^c^	60 (51-71.4)	76 (64.5-89)	63.8 (53-77)
CD4 count, median (IQR), cells/mm^3d^	413 (296-581)	363 (271-502)	399 (288-561)
CD4 category, no. (%), cells/mm^3d^			
200-350	9705 (37.4)	4019 (46.8)	13,724 (39.8)
>350	16224 (62.6)	4566 (53.2)	20790 (60.2)
ART naïve at enrollment, No. (%)	33345 (91.1)	11645 (90.6)	44990 (90.9)
WHO stage at enrollment, No. (%)^e^			
Stage 1	17304 (60.5)	4803 (48.0)	22107 (57.2)
Stage 2	6647 (23.2)	2658 (26.6)	9305 (24.1)
Stage 3	4666 (16.3)	2547 (25.5)	7213 (18.7)
Urban	16970 (46.4)	6165 (47.9)	23135 (46.8)
Married/living with partner^f^	19045 (54.0)	9127 (73.1)	28172 (59.0)

*Abbreviations*: *IQR* interquartile range; *SBP* systolic blood pressure; *DBP* diastolic blood pressure; *BMI* body mass index; *ART* antiretroviral therapy; *WHO*, World Health Organization. SI conversion factors: To convert hemoglobin to grams per liter, multiple by 10.

^a^n = 31882 for women; 11042 for men.

^b^n = 26,826 for women; 9099 for men.

^c^n = 25,570 for women; 8799 for men.

^d^n = 25,929 for women; 8585 for men.

^e^n = 28617 for women; 10008 for men.

^f^n = 47745.

Among women, there were 1351 deaths in 75,786 person years (py) of follow up (mortality rate 1.8/100 py). Among men, there were 966 deaths in 25,369 py of follow up (mortality rate 3.8/100 py). With respect to blood pressure, within gender, crude mortality rates are substantially higher for those in the lowest SBP and DBP categories compared to all other categories. The difference is particularly pronounced among men, where mortality rate is 10.7/100 py for those with SBP <100 and 16.4/100 py for those with DBP <60 mmHg.

**Figure 2:**
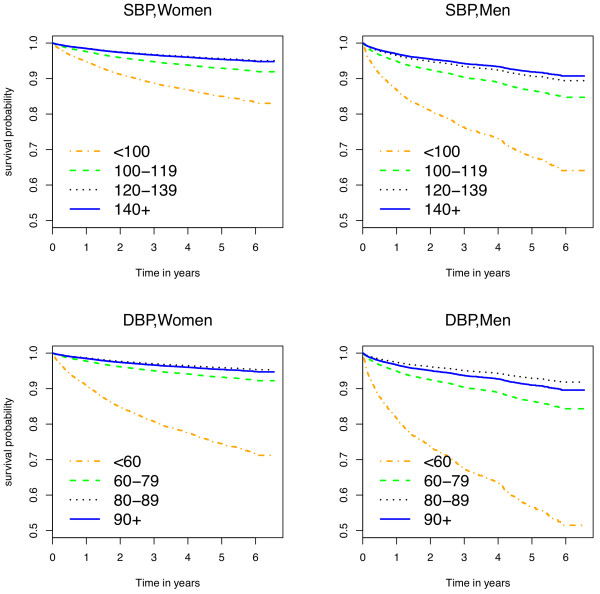
**Adjusted survival curves by SBP and DBP categories, stratified by gender.** Legend: Survival curves are adjusted for age (set to 35) and stratified by systolic and diastolic blood pressure categories. SBP: systolic blood pressure. DBP: diastolic blood pressure.

Figure 2 shows age-adjusted survival curves stratified by SBP and DBP categories, separately for each gender. The curves depict expected survival probabilities when age is fixed at 35 years demonstrating that those with low DBP and SBP have the highest age adjusted mortality rates. Additional files 2 and 3 (Figures S3 and S4) are smoothed linear spline curves applied to the unweighted sample of all individuals having baseline blood pressures showing the relationship between blood pressure and mortality rate among men and women with and without advanced HIV.

The unadjusted mortality rate was stratified according to the presence of advanced HIV disease and blood pressure in women and men (Table 2). The highest mortality rates were seen in those with the lowest SBP and DBP (systolic <100 mmHg 5.2/100 py [4.8-5.7]; diastolic <60 mmHg IR 9.2/100 py [8.3-10.2]). Patients with the lowest systolic and diastolic blood pressures also had lowest CD4 counts, body mass index and hemoglobin as shown in (Additional file 4: Table S4). In addition, the death rate among men without advanced disease and with SBP ≥140 mmHg (3.0, 95% CI: 1.6-5.5) was nearly three-fold greater than those with normal SBP (1.1, 95% CI: 0.7-1.7). This observation was not present among women or among those with advanced HIV disease. There was a similar relationship to high DBP such that high DBP in men without advanced HIV (2.9, 95% CI: 1.5-5.9) was associated with a higher death rate than those with normal DBP (1.7, 95% CI: 1.3-2.2).

**Table 2:** Unadjusted mortality rates per 100 person years by blood pressure groups for men and women with and without advanced HIV

**Characteristic**	**Person years**	**Women events**	**Mortality rate (95% CI)**	**Person years**	**Men events**	**Mortality rate (95% CI)**
**Advanced HIV**^**a**^						
Systolic blood pressure						
SBP <100 mmHg	3447	113	3.3 (2.7-3.9)	816	57	7.0 (5.4-9.1)
SBP 100-119 mmHg	18648	364	2.0 (1.8-2.2)	5966	219	3.7 (3.2-4.2)
SBP 120-139 mmHg	7954	116	1.5 (1.2-1.7)	4001	96	2.4 (2.0-2.9)
SBP ≥140 mmHg	1095	23	2.1 (1.4-3.2)	590	21	3.6 (2.3-5.5)
Diastolic blood pressure						
DBP <60 mmHg	1164	73	6.3 (5.0-8.0)	356	56	15.8 (12.1-20.5)
DBP 60-79 mmHg	23988	467	1.9 (1.8-2.1)	8142	283	3.5 (3.1-3.9)
DBP 80-89 mmHg	4913	58	1.2 (0.9-1.5)	2372	45	1.9 (1.4-2.5)
DBP ≥90 mmHg	1079	18	1.7 (1.1-2.6)	504	9	1.8 (0.9-3.4)
**Not advanced HIV**						
Systolic blood pressure						
SBP <100 mmHg	1707	28	1.6 (1.1-2.4)	221	9	4.1 (2.1-7.8)
SBP 100-119 mmHg	12625	91	0.7 (0.6-0.9)	2097	23	1.1 (0.7-1.7)
SBP 120-139 mmHg	6461	38	0.6 (0.4-0.8)	1906	34	1.8 (1.3-2.5)
SBP ≥140 mmHg	842	8	0.9 (0.5-1.9)	339	10	3.0 (1.6-5.5)
Diastolic blood pressure						
DBP <60 mmHg	824	21	2.5 (1.7-3.9)	89	2	2.2 (0.6-9.0)
DBP 60-79 mmHg	16301	121	0.7 (0.6-0.9)	3195	54	1.7 (1.3-2.2)
DBP 80-89 mmHg	3766	20	0.5 (0.3-0.8)	1006	12	1.2 (0.7-2.1)
DBP ≥90 mmHg	744	3	0.4 (0.1-1.3)	272	8	2.9 (1.5-5.9)

*Abbreviations*: *HIV* human immunodeficiency virus, *SBP* systolic blood pressure, *DBP* diastolic blood pressure.

^a^Advanced HIV is defined by CD4 count <350, or WHO Stage 2 or 3.

Table 3 shows results from the weighted proportional hazards regression model. For both men and women, SBP <100 or DBP <60 mmHg had greater mortality risk relative to those in the normotensive reference range. The effect of low DBP was most pronounced among those with more advanced HIV disease, with hazard ratio (HR) 2.76 (95% CI: 1.98-3.83) among women and 3.19 (95% CI: 2.14-4.74) among men. Both SBP 120-139 (HR: 1.91, 95% CI: 1.01-3.59) and ≥140 mmHg (HR: 2.39, 95% CI: 0.94-6.08) carry higher mortality risks among men whose HIV disease is not advanced relative to normotensives. This relationship was not observed among men with advanced HIV disease. High DBP was not associated with a greater risk of death. Low BMI, older age, higher creatinine, lower hemoglobin, an urban clinic setting, and not being married were all associated with greater risk of death regardless of HIV disease stage. Table 3 also demonstrates that the mortality risks associated with high blood pressure were generally smaller among patients with advanced HIV disease. We did not undertake significance testing of every pairwise comparison. In a separate analysis, the interaction between blood pressure level and HIV disease stage was found to be statistically significant for women (p < 0.001), and separately for men (p < 0.001).

**Table 3:** Mortality hazard ratios from proportional hazards regression models using inverse probability weighting for missing covariates

**Variable**	**Women**		**Men**	
	**Advanced HIV**^**a**^		**Advanced HIV**	
	**Yes**	**No**	**Yes**	**No**
**SBP, mmHg**				
<100	1.28 (0.98-1.68)	2.29 (1.37-3.80)	1.61 (1.09-2.39)	2.95 (1.17-7.44)
100-119	ref	ref	ref	ref
120-139	0.83 (0.64-1.07)	0.94 (0.60-1.47)	0.73 (0.53-1.00)	1.91 (1.01-3.59)
≥140	0.97 (0.58-1.60)	1.06 (0.47-2.41)	1.24 (0.74-2.08)	2.39 (0.94-6.08)
**DBP, mmHg**				
<60	2.76 (1.98-3.83)	2.26 (1.19-4.26)	3.19 (2.14-4.74)	1.88 (0.45-7.78)
60-79	ref	ref	ref	ref
80-89	0.65 (0.47-0.90)	0.67 (0.37-1.22)	0.68 (0.47-0.98)	0.92 (0.46-1.84)
≥90	1.15 (0.62-2.14)	0.57 (0.19-1.71)	0.66 (0.28-1.56)	1.65 (0.61-4.47)
**BMI, kg/m**^**2b**^				
<18.5	1.82 (1.55-2.13)			
18.5 - <25	ref			
25 - <30	0.90 (0.68-1.18)			
≥30	1.12 (0.67-1.88)			
**Age, years**				
<30	ref			
30-45	1.10 (0.92-1.31)			
46-60	1.28 (1.03-1.59)			
>60	2.11 (1.47-3.03)			
**Log creatinine**	1.28 (1.04-1.58)			
**Hemoglobin, g/dL**	0.81 (0.79-0.83)			
**ART naïve**	0.99 (0.67-1.45)			
**Urban clinic**	0.84 (0.73-0.98)			
**Married**	0.73 (0.64-0.85)			

*Abbreviations*: *SBP* systolic blood pressure, *BMI* body mass index, *ART* anti-retroviral therapy.

^a^Advanced HIV is defined as CD4 < 350, or WHO Stage 2 or 3.

^b^Hazard ratios for BMI, Age, log creatinine, hemoglobin, ART naïve, urban clinic and married apply to all 4 strata.

## Discussion and conclusions

Chronic, non-HIV related conditions are an increasingly important part of HIV management, a trend that has historically been underappreciated [19]. While the burden of high blood pressure among Africans living with HIV has been highlighted, the impact of blood pressure on important clinical endpoints has not been well established and therefore has not routinely been a focus of treatment in HIV programs in the region [12,20]. With this gap in the literature in mind, we have shown that low enrollment SBP or DBP is associated with a high mortality risk among HIV+ patients. There was also a relationship between higher initial SBP and mortality in men without advanced HIV. In adjusted analyses, the effect of high blood pressure was smaller than the effect of low blood pressure.

The greatest mortality risks in the present analysis were related to low blood pressure. In addition to advanced disease stage, other well-documented reasons for low blood pressure in HIV+ patients include adrenal insufficiency, autonomic dysfunction, concomitant infections, and the effects of bacterial translocation among others [21-23]. The cause of low blood pressure was not identifiable in this study, however, these factors as well as concomitant illness may have contributed to the observed low blood pressure. Lower blood pressure has also been observed in patients with more advanced HIV disease [24] and we posit this explanation in this analysis based on the deranged parameters of body mass index, hemoglobin and CD4 count in those with the lowest blood pressure. The mortality risk, however, was large and indicates the need to explore concomitant clinical factors as well as cause of death in this population. Such data and relatively simple measures such as blood pressure measurement are only recently beginning to be explored in HIV cohorts on the continent, highlighting a strength and relevance of this analysis [25].

Western literature supports a relationship between higher SBP and mortality in HIV+ men. In the Multicenter AIDS Cohort, Seaberg et al., demonstrated that the prevalence of systolic hypertension among men taking ART for less than two years was similar to that of HIV seronegative (-) men, but was nearly two-fold higher after two to five years of ART and five years or more of ART [26]. Other studies support the relationship between HIV positivity and higher blood pressure, but this is still debated [27-29]. Compared to Western countries, there has been relatively little attention paid to the overlap between non-communicable cardiovascular diseases and HIV in SSA. In a large systematic review and meta-analysis (50 studies including 38 from SSA) of early mortality in adults initiating ART in low- and middle-income countries, blood pressure was not reported as a covariate of interest in any of the studies [10].

Data from the SSA region regarding blood pressure, however, are slowly emerging. The overall rate of hypertension among HIV+ patients in SSA is estimated to be 8-19%, and slightly higher among men compared to women [12]. In Kenya, a program showed that when people are screened for HIV and non-communicable diseases simultaneously, HIV+ people had higher rates of hypertension (32%) than those who were HIV- (19%) [30]. Similar findings are reported from Tanzania, Botswana, and Nigeria while in our analysis hypertension was present in less than 10% of the population [31-33]. A systematic review and meta-analysis of the association between HIV and cardiometabolic traits, however, suggests that HIV+ patients have, on average, lower blood pressure than their HIV- counterparts [34]. It is noteworthy that the studies included in this review vary in patient population, method of screening, selection of patients and definitions of outcomes resulting in an unresolved relationship between blood pressure and HIV. Given the size of our HIV care program, we believe the observed relationship between high blood pressure and mortality may be generalizable to the region, however, country-specific data comparing HIV+ to HIV- patients are needed to put the magnitude of the issue in a relevant context. Both high and low blood pressure impact mortality in these patients suggesting that addressing blood pressure in HIV+ patients in SSA is potentially a lost opportunity to modify health and impact mortality.

The differences in mortality risk between men and women warrant further exploration. The reasons for the observed higher mortality risk related to high blood pressure among men compared to women may be related to a true sex-related difference in the impact of high blood pressure or reflect other factors that vary according to sex including less health-seeking behavior among men or higher burden of other cardiovascular risk factors (e.g., tobacco smoking) among men [35]. Moreover, most of the women in this analysis were of pre-menopausal age and may therefore carry lower risk of cardiovascular disease than men. Prospective analyses that can control for known sex-based differences will be able to elucidate whether higher blood pressure among HIV+ men carries a greater mortality risk than among women.

Some limitations of our study should be considered. This was a retrospective analysis of clinical data and variables, including blood pressure, that were not measured routinely in every patient or in a standard, protocol-driven manner. Our analysis is therefore limited by a selection bias potentially introduced at the time of the clinical encounter and our observations do not confirm causality. We had a significant proportion of patients missing data and while this is often the situation in reality, we addressed this by using IPW to account for differences in mortality between those with and without missing covariates. Our weighting approach resulted in similar survival distributions between those with fully observed and partially observed covariates and supported its use for fitting the proportional hazards models. Incomplete data is not uncommon in HIV treatment programs in SSA and our approach represents one method for addressing this phenomenon [11]. Information on diabetes, smoking or use of cardiovascular medications was also not routinely measured during this time. Further, cause of death information was not available for this cohort and we are therefore limited in our ability to comment on the mechanism of the association between BP and mortality in this analysis. Given than non-communicable cardiovascular causes of death present at a younger age in populations earlier in the epidemiologic transition, it seems necessary to investigate these associations as well as possible mechanisms in HIV+ patients. Data from the region using verbal autopsy, however, show that non-communicable diseases account for between 2 and 10% of deaths among HIV+ individuals on ART [36,37].

In summary, while cardiovascular risk factor levels remain low in SSA in general and life expectancy among HIV+ individuals nears 80% of normal, a rare opportunity currently exists to understand and decrease or prevent cardiovascular risk in a population still on the cusp of a non-communicable disease epidemic. Literature from developed countries support a relationship between HIV and cardiovascular disease risk and the burden of HIV in SSA calls for more attention to these comorbidities. Using clinical data from a large HIV treatment program in western Kenya, we have demonstrated that low and high blood pressures in HIV+ patients are important predictors of mortality with low blood pressure carrying the greatest mortality risk. Of particular clinical relevance is the finding that high blood pressure is associated with higher mortality risk among patients whose HIV disease is not advanced. Therapy for high blood pressure should be instituted when indicated. Patients with low blood pressure should undergo a thorough assessment to uncover and reverse the causes. To our knowledge, this is one of the first and largest reports from the region indicating this relationship and highlights the need to address cardiovascular diseases in this population, especially among patients without advanced HIV. Programs in SSA that are narrowly focused on HIV treatment alone may be missing an important opportunity to modify health.

## Abbreviations

AIDS: Acquired immune deficiency syndrome; ART: Anti-retroviral therapy; BMI: Body mass index; DBP: Diastolic blood pressure; HIV: Human immunodeficiency virus; HIV+: Human immunodeficiency virus seropositive; HIV-: Human immunodeficiency virus seronegative; HR: Hazard ratio; IPW: Inverse probability weighting; SBP: Systolic blood pressure; SSA: Sub-Saharan Africa; WHO: World Health Organization.

## Competing interest

The authors declare that they have no competing interests.

## Authors’ contributions

GSB participated in the study design, data interpretation and wrote the first draft of the manuscript. JWH participated in study design, data analysis, data interpretation and wrote the technical appendix. AK participated in data analysis and data interpretation. TLH participated in data interpretation. ES contributed to data acquisition. SK participated in the study design, data acquisition and data interpretation. EJV participated in the study design and data interpretation. All authors contributed to drafting or revising the manuscript critically for important intellectual content, and gave final approval of the version to be published. All authors had full access to all of the data in the study and take responsibility for the integrity of the data and the accuracy of the data analysis.

## Pre-publication history

The pre-publication history for this paper can be accessed here:

http://www.biomedcentral.com/1471-2334/14/284/prepub

## Supplementary Material

Additional file 1 **Technical appendix.** Supplementary technical appendix. The supporting technical methodological appendix includes the detailed description of our inverse probability weighting approach. Additional file 1 includes **Tables S1**, **S2** and **S3**; and **Figures S1** and **S2**. Click here for file

Additional file 2: Figure S3 The panels in **Figure S3** are smoothed spline curves, applied to the unweighted sample of all individuals having baseline systolic and diastolic blood pressures, displaying the relationship between blood pressure and log hazard ratio among men. The figure displays the relationships between log mortality hazard ratio and **(A)** systolic blood pressure in men with advanced HIV, **(B)** diastolic blood pressure in men with advanced HIV, **(C)** systolic blood pressure in men without advanced HIV and **(D)** diastolic blood pressure in men without advanced HIV. Click here for file

Additional file 3: Figure S4 The panels in **Figure S4** are smoothed spline curves, applied to the unweighted sample of all individuals having baseline systolic and diastolic blood pressures, displaying the relationship between blood pressure and log hazard ratio among women. The figure displays the relationships between log mortality hazard ratio and **(A)** systolic blood pressure in women with advanced HIV, **(B)** diastolic blood pressure in women with advanced HIV, **(C)** systolic blood pressure in women without advanced HIV and **(D)** diastolic blood pressure in women without advanced HIV. Click here for file

Additional file 4: Table S4 Supplementary table describing the distribution of CD4 count, body mass index and hemoglobin stratified by blood pressure level. Click here for file
