# Supplementary material for: Blood pressure level impacts risk of death among HIV seropositive adults in Kenya: a retrospective analysis of electronic health records
Source: BMC Infect Dis. 2014 May 22;14:284. doi: 10.1186/1471-2334-14-284 (PMC4046023; doi:10.1186/1471-2334-14-284)
Supplement: Additional file 1 — Technical appendix. Supplementary technical appendix. The supporting technical methodological appendix includes the detailed description of our inverse probability weighting approach. Additional file 1 includes Tables S1, S2 and S3; and Figures S1 and S2. [file 1471-2334-14-284-S1.docx]

**Additional File 1. Supplementary technical appendix**

Prepared by Joseph W. Hogan

**1. Notation and model specification**

Inverse probability weighting is used for correction of potential biases due to missing covariates. Define the following. For each individual, we observe the pair (*T*, **Δ**), where *T* is the observed follow up time from baseline, and **Δ** is an indicator of whether *T* is a death time. Specifically, if the actual death time is *U,* then **Δ** = 1 if *T* = *U* and **Δ** = 0 if *T* < *U.*

In addition, we observe several covariates on each individual. The main covariate of interest is the pair (S, *D),* where *S* denotes systolic blood pressure (SBP) and *D* is diastolic blood pressure (DBP). Each of these is a four-level categorical variable as described in the paper. Key stratification variables are gender and severity of HIV disease. Gender is denoted by *G* (1 if male, 0 if female). Using CD4 category and WHO stage, we formed a new covariate *H* to denote HIV severity, where *H* = 1 if WHO stage is 2 or 3, or if CD4 count is less than or equal to 350, and *H* = 0 otherwise.

In addition to covariates on blood pressure, gender, and HIV severity, there are several additional covariates that we denote as *X* = (X_F_,X_P_), where X_F_ denotes the subset of covariates that are fully observed for each individual, and X_P_ are the subset of covariates that are only partially observed (i.e., where at least one covariate is unobserved for at least one person). In our analysis, the components of X_F_ are SBP, DBP, age at baseline, antiretroviral therapy (ART) status at baseline (naïve or not), and clinic location (urban/rural). Components of X_P_ are BMI, marital status (married vs. not), hemoglobin level, and creatinine level.

The objective of the regression modeling is to characterize the relationship between mortality and baseline blood pressure, conditional on (adjusting for) baseline covariates in *X.* This relationship is captured using the proportional hazards regression model

log{λ(t)} = log{λ_0_(t)}+ *f(S,D,H,G;α,β)+Xθ,*

where λ(t) is the hazard of death at time *t,* λ_0_(t) is a baseline hazard function, the term *f(S,D,H,G; α,β)* captures the joint effect of BP, gender and HIV on the hazard of mortality, and *θ* contains the coefficients (log hazard ratios) corresponding to the effects of covariates in *X.*

The function *f* (S, *D, H, G; α,β)* is constructed so that there are separate SBP and DBP effects within each of the four strata defined by gender and HIV severity. Specifically,

*f(S,D,H,G; α,β)* = *GH(α_11_S+β_11_D)*

+ *G(1-H)( α_10_S* + *β_10_D)*

+ (1-*G)H(α_01_S* + *β_01_D)*

+ (1-*G)(1-H)( α_00_S* + *β_00_D).*

Hence the parameters *α_gh_* and *β_gh_* correspond, respectively, to log hazard ratios for SBP and DBP within strata where *G* = *g* and *H* = *h.* For example *α_01_* corresponds to the log hazard ratios for SBP effect among females with severe HIV disease (G = 0, *H* = 1).

The parameters of this model index the *full data,* not the subset of data having complete covariate information. The term *Xθ,* which captures effects of other covariates, includes the effects of both fully and partially observed covariates, and can be decomposed as *Xθ* = X_F_*θ_1_* + X_P_*θ_2_.*

**2. Creating a weighted sample for reducing selection bias**

**2.1 Diagnosing potential selection biases**

One approach to analysis of data with incomplete covariates is to use only those individuals with complete information. In our sample, 22,353 of the 49,475 individuals have complete covariate information. Unless those with complete information are a random draw from the full sample, this approach leaves open the possibility of selection bias, whereby those with missing covariates have different expected mortality than those with complete covariates. Indeed, Supplementary Figure S1 indicates that survival distributions are much different, even within categories of DBP and SBP. For the unweighted sample, it is evident that those with missing covariates tend to have higher mortality rates (lower survival rates). The discrepancy is particularly pronounced among those with SBP <100 and DBP <60 mmHg.

**2.2 Inverse probability weighting for reducing selection bias**

One method for handling selection bias is the use of inverse probability weighting, or IPW. The diagnostic plots described above suggest that if we use only those with complete covariate information to fit our regression model, both the mortality rates and the effects of SBP and DBP may be subject to selection biases.

The IPW method is used to calculate individual-specific sampling weights that reflect the differential probability of being included in the analysis sample (i.e., having complete covariate information). Under certain assumptions about how the sample selection depends on survival time and on other covariates, we can reduce or even largely eliminate this source of bias by fitting the regression model to the weighted data. Our analysis relies on the assumption that the probability of having missing covariates can be fully explained by those covariates in X_F_ that are completely observed, and by the information on mortality encoded by (*T*, **Δ**).

Formally, the method proceeds as follows. Let *R* denote the sample selection indicator, with *R* = 1 if all elements of *X* are observed, and *R* = 0 if one or more is missing. Our analysis relies on three key assumptions: that the selection mechanism depends on observed information (*T*, **Δ**, X_F_), but conditionally on these, does not depend on covariates in X_P_ that are potentially missing; that the probability of having incomplete covariates can be consistently estimated using a function of the observed information (i.e., using predicted sampling probabilities from a logistic regression); and that all individuals are potentially prone to having missing covariates. More detail about these assumptions, which are common in the analysis of incomplete data, have been published [1-3].

**2.3 Implementation**

Based on these assumptions, we used a logistic regression to derive sampling weights. The selection probability is denoted by *p* = Pr(R = 1 | X_F_*, T*, **Δ**). Notice that sample selection depends on the actual survival time *U* through observed information about survival as encoded by (*T*, **Δ**). Hence the selection weights explicitly model the association between sample selection and survival time, so that the weighted dataset will alleviate bias due to differential survival between those with and without missing covariates.

The linear predictor of the logistic regression model includes main effects of both *T*, **Δ** and the following components of X_F_*:* SBP (4 categories); DBP (4 categories); age, categorized as (18,30], (30,45], (45,60], and (60, ∞); urban clinic (yes/no); ART naïve at baseline (yes/no), and gender (male/female). Follow up time *T* is categorized in years as (0, 1/12], (1/12, 1/2], (1/2, 1], [1, 2), [2,3), [3, ∞). It also includes interactions **Δ** × SBP, **Δ** × DBP, *T* × SBP, and *T* × DBP. The model is fit to the full sample of 49,475 individuals; estimated regression coefficients are given in Supplementary Table S1. Predicted probabilities from this model are denoted by $\hat{p}$*.*

We also fit a logistic regression model for *P(R* = 1 | *V);* predicted values from this model can be used to stabilize the weights [2]. The components of *V* used in this model are SBP, DBP, age (categorized as above), gender, urban clinic indicator, and ART naïve indicator. Only main effects are included in the linear predictor; the estimated regression coefficients appear in Supplementary Table S2. The predicted values from this model are denoted by $\hat{q}$*.* Finally, for each individual, we generate a stabilized sampling weight $\hat{w}$ = $\hat{q}$*/*$\hat{p}$ for those with *R* = 1 and $\hat{w}$ = (1-$\hat{q}$*)/(1-* $\hat{p}$*)* for those with *R* = 0.

Checking the distribution of weights is an important component of using the IPW method. Estimates and inferences from weighted data can be susceptible to very large weights. Supplementary Table S3 summarizes the distribution of weights for the analysis sample (those with *R* = 1). Referring to the list of largest and smallest weights, we see that outlying or extreme weights do not present a major concern.

To assess whether the weighting corrects the selection bias, we compare weighted survival curves between those with *R* = 1 and *R* = 0. We stratify on SBP and DBP as described above. Referring to Supplementary Figure S2, we see that the survival distributions between those with fully observed and partially observed covariates is much more similar in the weighted sample, suggesting that a substantial amount of selection bias is reduced in the weighted sample, and supporting the use of inverse probability weighting for fitting the proportional hazards regression reported in Table 3 of the paper. That model is fit to those with *R* = 1, using weights $\hat{q}$*/*$\hat{p}$*.*

**Supplementary Table S1. Coefficient estimates for sample selection model fit to full sample of 49,475 observations.**

| **R** | **Coef.** | **Std. Err.** | **z** | **P>\|z\|** | **[95% Conf. Interval]** | |
| --- | --- | --- | --- | --- | --- | --- |
| **_d#dbp_cat** |  |  |  |  |  |  |
| 0 2 | .0850482 | .0936623 | 0.91 | 0.364 | -.0985266 | .2686229 |
| 0 3 | .0453626 | .1003104 | 0.45 | 0.651 | -.1512422 | .2419675 |
| 0 4 | .0670795 | .1258584 | 0.53 | 0.594 | -.1795984 | .3137573 |
| 1 1 | -.1686927 | .3234862 | -0.52 | 0.602 | -.802714 | .4653286 |
| 1 2 | .0776457 | .2851011 | 0.27 | 0.785 | -.4811423 | .6364337 |
| 1 3 | .1503151 | .3102742 | 0.48 | 0.628 | -.4578112 | .7584413 |
| 1 4 | 0 | (omitted) |  |  |  |  |
| **_d#sbp_cat** |  |  |  |  |  |  |
| 0 2 | .0568029 | .0606391 | 0.94 | 0.349 | -.0620476 | .1756534 |
| 0 3 | .1507723 | .06491 | 2.32 | 0.020 | .0235511 | .2779935 |
| 0 4 | -.1144233 | .1047691 | -1.09 | 0.275 | -.3197669 | .0909203 |
| 1 1 | -.8127495 | .284285 | -2.86 | 0.004 | -1.369938 | -.2555612 |
| 1 2 | -.702082 | .262753 | -2.67 | 0.008 | -1.217068 | -.1870956 |
| 1 3 | -.4967586 | .2710706 | -1.83 | 0.067 | -1.028047 | .0345301 |
| 1 4 | 0 | (omitted) |  |  |  |  |
| **time_cat#sbp_cat** |  |  |  |  |  |  |
| 0 2 | .2324156 | .1331088 | 1.75 | 0.081 | -.0284729 | .4933041 |
| 0 3 | .1442543 | .1468719 | 0.98 | 0.326 | -.1436094 | .4321179 |
| 0 4 | .3052862 | .2518816 | 1.21 | 0.226 | -.1883927 | .7989651 |
| 1 2 | .1167034 | .1070184 | 1.09 | 0.275 | -.0930488 | .3264556 |
| 1 3 | .1786182 | .1159947 | 1.54 | 0.124 | -.0487272 | .4059637 |
| 1 4 | .4638042 | .1874854 | 2.47 | 0.013 | .0963396 | .8312688 |
| 2 2 | .0103935 | .1105477 | 0.09 | 0.925 | -.206276 | .227063 |
| 2 3 | -.05855 | .1175244 | -0.50 | 0.618 | -.2888936 | .1717935 |
| 2 4 | .3854119 | .181862 | 2.12 | 0.034 | .028969 | .7418548 |
| 3 2 | -.0602441 | .0989141 | -0.61 | 0.542 | -.2541121 | .1336239 |
| 3 3 | -.0877936 | .1052269 | -0.83 | 0.404 | -.2940345 | .1184473 |
| 3 4 | .4372793 | .1546384 | 2.83 | 0.005 | .1341936 | .740365 |
| 4 2 | .0170947 | .1093614 | 0.16 | 0.876 | -.1972498 | .2314392 |
| 4 3 | -.0475768 | .1161477 | -0.41 | 0.682 | -.275222 | .1800685 |
| 4 4 | .2249449 | .177105 | 1.27 | 0.204 | -.1221745 | .5720644 |
| 5 2 | 0 | (omitted) |  |  |  |  |
| 5 3 | 0 | (omitted) |  |  |  |  |
| 5 4 | 0 | (omitted) |  |  |  |  |
| **time_cat#dbp_cat** |  |  |  |  |  |  |
| 0 2 | .3092407 | .2004798 | 1.54 | 0.123 | -.0836925 | .702174 |
| 0 3 | .3940955 | .2214005 | 1.78 | 0.075 | -.0398414 | .8280325 |
| 0 4 | .4105019 | .2998368 | 1.37 | 0.171 | -.1771674 | .9981713 |
| 1 2 | .2066877 | .1481936 | 1.39 | 0.163 | -.0837665 | .4971419 |
| 1 3 | .3737058 | .1650525 | 2.26 | 0.024 | .0502088 | .6972027 |
| 1 4 | .1937169 | .2206907 | 0.88 | 0.380 | -.2388289 | .6262626 |
| 2 2 | .3094939 | .1585043 | 1.95 | 0.051 | -.0011689 | .6201566 |
| 2 3 | .3345194 | .1727367 | 1.94 | 0.053 | -.0040383 | .673077 |
| 2 4 | .1188229 | .2264271 | 0.52 | 0.600 | -.3249661 | .5626119 |
| 3 2 | .0235595 | .1429916 | 0.16 | 0.869 | -.2566989 | .3038179 |
| 3 3 | -.0451778 | .1548016 | -0.29 | 0.770 | -.3485834 | .2582277 |
| 3 4 | -.0031648 | .2040232 | -0.02 | 0.988 | -.403043 | .3967134 |
| 4 2 | -.1492781 | .1526164 | -0.98 | 0.328 | -.4484007 | .1498445 |
| 4 3 | -.1780031 | .1657219 | -1.07 | 0.283 | -.502812 | .1468058 |
| 4 4 | -.0724626 | .2157015 | -0.34 | 0.737 | -.4952297 | .3503045 |
| 5 2 | 0 | (omitted) |  |  |  |  |
| 5 3 | 0 | (omitted) |  |  |  |  |
| 5 4 | 0 | (omitted) |  |  |  |  |
| **age_cat** |  |  |  |  |  |  |
| 1 | .0563183 | .0215993 | 2.61 | 0.009 | .0139846 | .0986521 |
| 2 | .1191346 | .0302873 | 3.93 | 0.000 | .0597725 | .1784967 |
| 3 | .0136761 | .0698817 | 0.20 | 0.845 | -.1232895 | .1506417 |
|  |  |  |  |  |  |  |
| _d | .3365233 | .3477147 | 0.97 | 0.333 | -.344985 | 1.018032 |
| **time_cat** |  |  |  |  |  |  |
| 1 | 1.631059 | .2166226 | 7.53 | 0.000 | 1.206487 | 2.055632 |
| 2 | 2.064133 | .2265415 | 9.11 | 0.000 | 1.62012 | 2.508146 |
| 3 | 2.422256 | .2147192 | 11.28 | 0.000 | 2.001414 | 2.843098 |
| 4 | 2.116021 | .2252682 | 9.39 | 0.000 | 1.674503 | 2.557538 |
| 5 | 2.386886 | .2046103 | 11.67 | 0.000 | 1.985858 | 2.787915 |
| **urban** | .2389862 | .0191779 | 12.46 | 0.000 | .2013982 | .2765743 |
| **arv_naive** | 1.089565 | .0363024 | 30.01 | 0.000 | 1.018413 | 1.160716 |
| **male** | -.1404703 | .0224257 | -6.26 | 0.000 | -.1844238 | -.0965168 |
| **_cons** | -3.504464 | .1814207 | -19.32 | 0.000 | -3.860042 | -3.148886 |

**Supplementary Table S2. Coefficient estimates for stabilization factor model, fit to full sample of 49,475 observations.**

| **R** | **Coef.** | **Std. Err.** | **z** | **P>\|z\|** | **[95% Conf. Interval]** | |
| --- | --- | --- | --- | --- | --- | --- |
| **sbp_cat** |  |  |  |  |  |  |
| 2 | .1876924 | .032061 | 5.85 | 0.000 | .1248541 | .2505308 |
| 3 | .2592566 | .0344568 | 7.52 | 0.000 | .1917225 | .3267907 |
| 4 | .3262454 | .0532363 | 6.13 | 0.000 | .2219042 | .4305866 |
| **dbp_cat** |  |  |  |  |  |  |
| 2 | .3042064 | .0452287 | 6.73 | 0.000 | .2155597 | .3928531 |
| 3 | .2696373 | .0496901 | 5.43 | 0.000 | .1722464 | .3670282 |
| 4 | .2631915 | .0663557 | 3.97 | 0.000 | .1331367 | .3932463 |
| **age_cat** |  |  |  |  |  |  |
| 1 | .157803 | .0206003 | 7.66 | 0.000 | .1174272 | .1981789 |
| 2 | .2450902 | .0290644 | 8.43 | 0.000 | .188125 | .3020554 |
| 3 | .1098214 | .0672573 | 1.63 | 0.102 | -.0220005 | .2416434 |
| **male** | -.1872858 | .0215726 | -8.68 | 0.000 | -.2295674 | -.1450043 |
| **urban** | .2598522 | .0183982 | 14.12 | 0.000 | .2237923 | .2959121 |
| **arv_naive** | .9576382 | .0356182 | 26.89 | 0.000 | .8878277 | 1.027449 |
| **_cons** | -1.739211 | .061715 | -28.18 | 0.000 | -1.86017 | -1.618251 |

**Supplementary Table S3. Summary of the distribution of inverse probability weights for the 22,353 observations without missing covariate values.** The table indicates that the smallest weight is .6 and the largest is 5.4.

| **IPW_alt** | | | | |
| --- | --- | --- | --- | --- |
|  | **Percentiles** | **Smallest** |  |  |
| **1%** | .7238264 | .6007009 |  |  |
| **5%** | .7774343 | .606235 |  |  |
| **10%** | .8077483 | .6143939 | Obs | 22353 |
| **25%** | .838657 | .6256607 | Sum of Wgt. | 22353 |
|  |  |  |  |  |
| **50%** | .876625 |  | Mean | 1.000152 |
|  |  | **Largest** | Std. Dev. | .4486482 |
| **75%** | 1.01046 | 4.839532 |  |  |
| **90%** | 1.079347 | 4.961116 | Variance | .2012852 |
| **95%** | 1.379468 | 5.341346 | Skewness | 4.176033 |
| **99%** | 3.059055 | 5.378972 | Kurtosis | 20.74859 |

**Supplementary Figure S1. Comparison of survival distributions by SBP and DBP categories using unweighted sample.** R=1 denotes sample with complete covariates (n=22,353) and R=0 denotes sample with incomplete covariates (n=27,122).


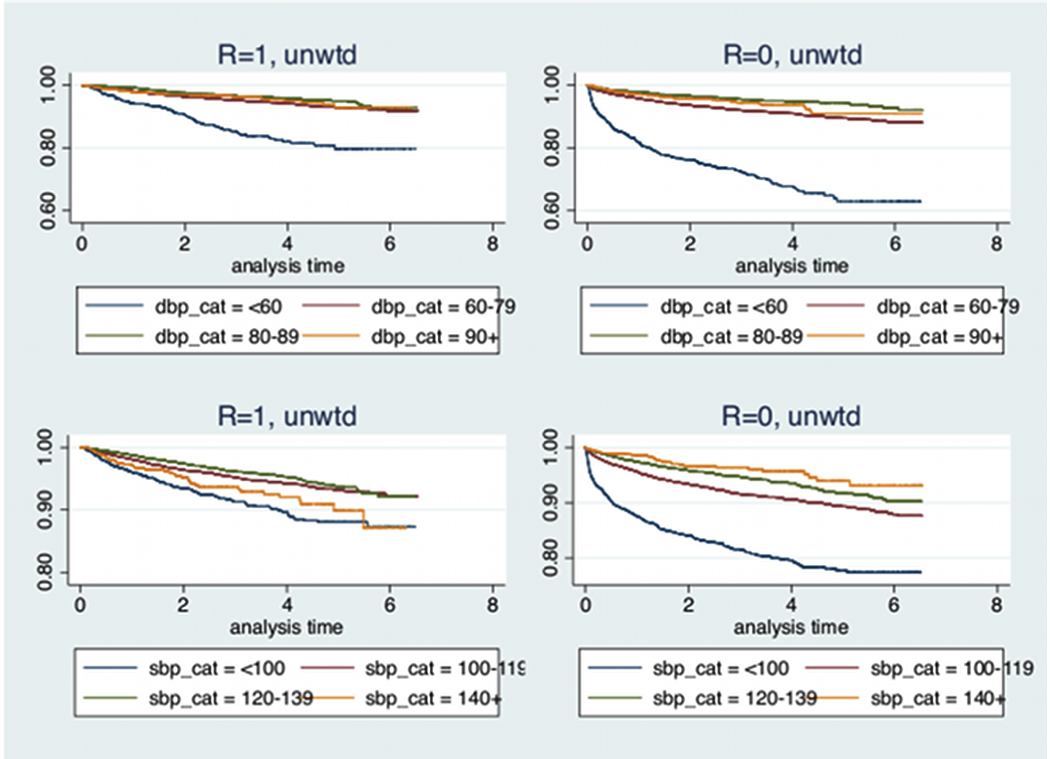


**Supplementary Figure S2. Comparison of survival distributions by SBP and DBP categories using weighted sample.** R=1 denotes sample with complete covariates (n=22,353) and R=0 denotes sample with incomplete covariates (n=27,122). Compared to the unweighted sample, the survival distributions are more similar between those with fully observed (R=1) and partially observed (R=0) covariates.


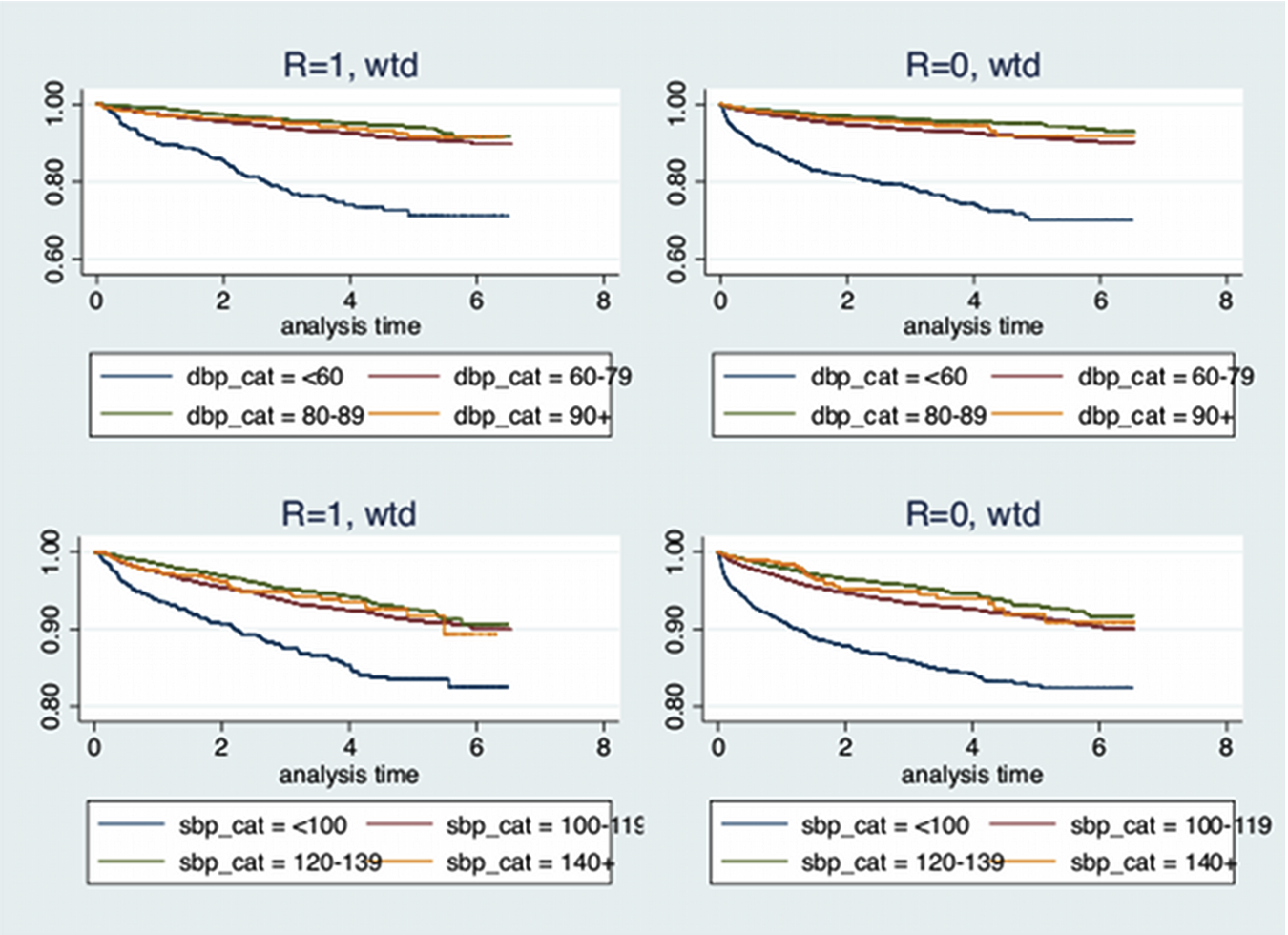


**Supplementary References**

1. Wang C, Chen H. Augmented inverse probability weighted estimator for Cox missing covariate regression. Biometrics, **2001**; 57: 414-419.

2. Hernan M, Brumback B, Robins J. Marginal structural models to estimate the joint causal effect of nonrandomized treatments. J Am Stat Assoc, **2001**; 96: 440-448.

3. Rubin D. Inference and missing data. Biometrika, **1976**; 63: 581-590.
