# Supplementary figures and images for: Blood pressure level impacts risk of death among HIV seropositive adults in Kenya: a retrospective analysis of electronic health records
Source: BMC Infect Dis. 2014 May 22;14:284. doi: 10.1186/1471-2334-14-284 (PMC4046023; doi:10.1186/1471-2334-14-284)

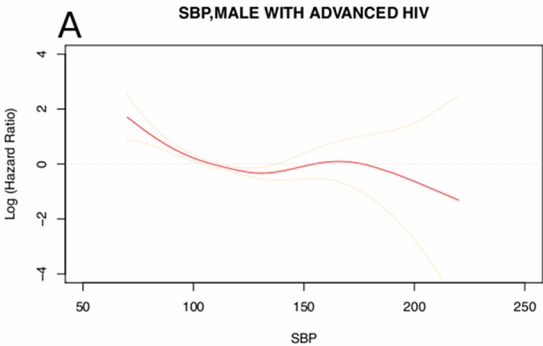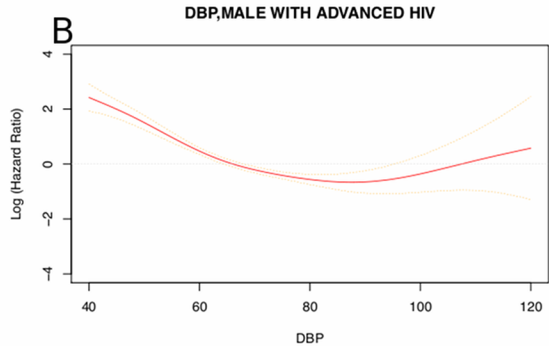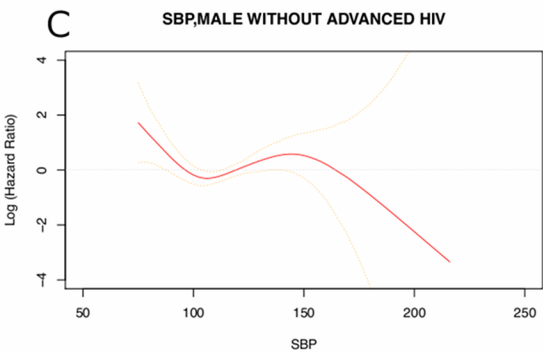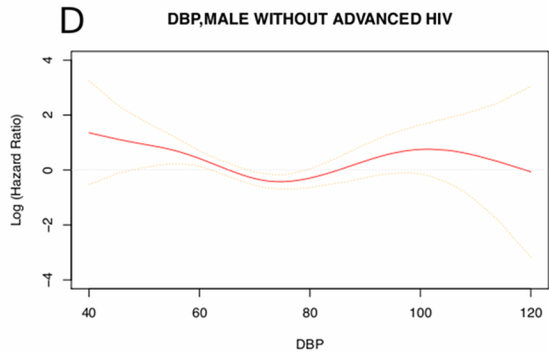

Supplement: Additional file 2: Figure S3 — The panels in Figure S3 are smoothed spline curves, applied to the unweighted sample of all individuals having baseline systolic and diastolic blood pressures, displaying the relationship between blood pressure and log hazard ratio among men. The figure displays the relationships between log mortality hazard ratio and (A) systolic blood pressure in men with advanced HIV, (B) diastolic blood pressure in men with advanced HIV, (C) systolic blood pressure in men without advanced HIV and (D) diastolic blood pressure in men without advanced HIV. [file 1471-2334-14-284-S2.pdf]

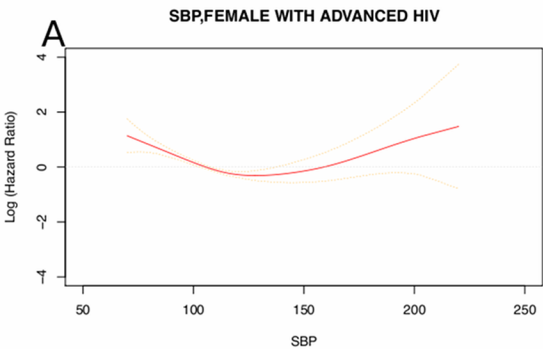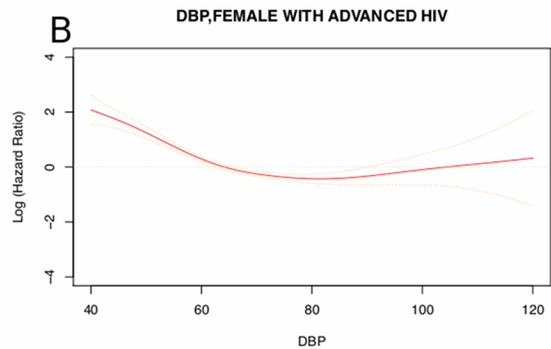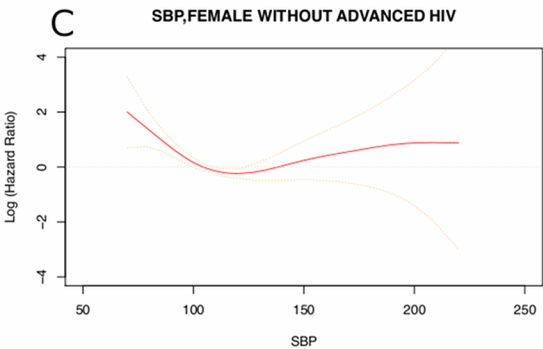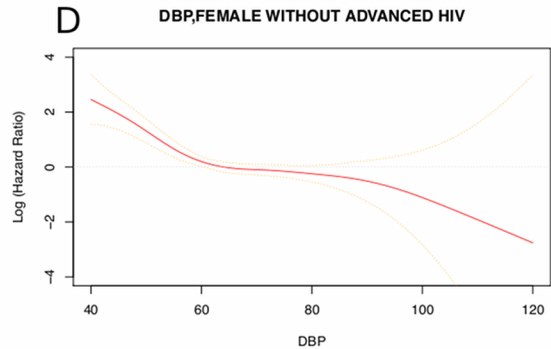

Supplement: Additional file 3: Figure S4 — The panels in Figure S4 are smoothed spline curves, applied to the unweighted sample of all individuals having baseline systolic and diastolic blood pressures, displaying the relationship between blood pressure and log hazard ratio among women. The figure displays the relationships between log mortality hazard ratio and (A) systolic blood pressure in women with advanced HIV, (B) diastolic blood pressure in women with advanced HIV, (C) systolic blood pressure in women without advanced HIV and (D) diastolic blood pressure in women without advanced HIV. [file 1471-2334-14-284-S3.pdf]
