# Supplementary material for: Blood pressure level impacts risk of death among HIV seropositive adults in Kenya: a retrospective analysis of electronic health records
Source: BMC Infect Dis. 2014 May 22;14:284. doi: 10.1186/1471-2334-14-284 (PMC4046023; doi:10.1186/1471-2334-14-284)
Supplement: Additional file 4: Table S4 — Supplementary table describing the distribution of CD4 count, body mass index and hemoglobin stratified by blood pressure level. [file 1471-2334-14-284-S4.pdf]

Additional File 4. Supplementary Table S4

Supplementary Table S4. CD4 count, body mass index and hemoglobin distribution stratified by blood pressure level

Characteristic	SBP<100 or DBP<60 mmHg Median (IQR) or n(%)	SBP 100-139 or DBP 60-89 mmHg Median (IQR) or n(%)	SBP>=140 or DBP>=90 mmHg Median (IQR) or n(%)
**No. of observations**	6480 (13%)	39340 (80%)	3655 (7%)
**CD4 count (cells/mm^3)**	371 (272-558)	404 (291-567)	397 (289-558)
**Body mass index (kg/m^2)**	19.7(17.7-21.9)	21.2 (19.2-23.5)	22.5 (20.2-25.6)
**Hemoglobin (g/dL)**	11.2 (9.6-12.8)	12.2 (10.6-13.7)	13 (11.3-14.4)

Abbreviations: SBP, systolic blood pressure; DBP, diastolic blood pressure
